# Synthesis and evaluation of gene delivery vectors based on PEI-modified metal-organic framework (MOF) nanoparticles

**DOI:** 10.22038/IJBMS.2023.71892.15644

**Published:** 2024

**Authors:** Somayeh Khosrojerdi, Leila Gholami, Majid Khazaei, Alireza Hashemzadeh, Majid Darroudi, Reza Kazemi Oskuee

**Affiliations:** 1 Department of Medical Biotechnology and Nanotechnology, Faculty of Medicine, Mashhad University of Medical Sciences, Mashhad, Iran; 2 Department of Molecular Medicine, Faculty of Advanced Technologies in Medicine, Iran University of Medical Sciences (IUMS), Tehran, Iran; 3 Metabolic Syndrome Research Center, Mashhad University of Medical Sciences, Mashhad, Iran; 4 Targeted Drug Delivery Research Center, Institute of Pharmaceutical Technology, Mashhad University of Medical Sciences, Mashhad, Iran; 5 Department of Basic Medical Sciences, Neyshabur University of Medical Sciences, Neyshabur, Iran; 6 Applied Biomedical Research Center, Mashhad University of Medical Sciences, Mashhad, Iran

**Keywords:** Gene delivery, Metal-organic frameworks, Polyethyleneimine, Transfection, UIO-66

## Abstract

**Objective(s):**

Zirconium-based metal-organic frameworks (MOFs) nanostructures, due to their capability of easy surface modification, are considered interesting structures for delivery. In the present study, the surfaces of UIO-66 and NH2-UIO-66 MOFs were modified by polyethyleneimine (PEI) 10000 Da, and their efficiency for plasmid delivery was evaluated.

**Materials and Methods:**

Two different approaches, were employed to prepare surface-modified nanoparticles. The physicochemical characteristics of the resulting nanoparticles, as well as their transfection efficiency and cytotoxicity, were investigated on the A549 cell line.

**Results:**

The sizes of DNA/nanocarriers for PEI-modified UIO-66 (PEI-UIO-66) were between 212–291 nm and 267–321 nm for PEI 6-bromohexanoic acid linked UIO-66 (PEI-HEX-UIO-66). The zeta potential of all was positive with the ranges of +16 to +20 mV and +23 to +26 mV for PEI-UIO-66 and PEI-HEX-UIO-66, respectively. Cellular assay results showed that the PEI linking method had a higher rate of gene transfection efficiency with minimal cytotoxicity than the wet impregnation method. The difference between transfection of modified nanoparticles compared to the PEI 10 kDa was not significant but the PEI-HEX-UIO-66 showed less cytotoxicity.

**Conclusion:**

The present study suggested that the post-synthetic modification of MOFs with PEI 10000 Da through EDC/NHS+6-bromohexanoic acid reaction can be considered as an effective approach for modifying MOFs’ structure in order to obtain nanoparticles with better biological function in the gene delivery process.

## Introduction

Gene delivery has shown great promise for treating a wide range of diseases such as cancer, cystic fibrosis, diabetes, familial hypercholesterolemia, heart disease, hemophilia, and AIDS (1-4). A successful clinical application of gene-based therapy highly depends on an efficient gene delivery system; therefore, the development of safe and effective vectors for gene delivery is a major hurdle in the application of these carriers in the clinical setting (1).

The viral vectors such as retroviruses, lentiviruses, and adenoviruses owned the largest share in gene therapy clinical trials (5, 6). Despite their efficiency, usage of these vectors is limited due to their intrinsic drawbacks comprising broad tropism, limited DNA packaging capacity (5), insertion mutation (7), carcinogenesis (5, 8), difficulty in production (9), immunogenicity (5), generalized toxicity (10), and other side effects (7). 

The non-viral vectors are synthetic or natural compounds such as cationic polymers, cationic liposomes, silica, and carbon tubes (7, 11). Their different properties, including their ability to carry payloads in different sizes, low toxicity and immunogenic response (12), low cost (11), and easy production, make them good candidates for gene delivery studies (11), also, with the successful usage of mRNA vaccines to treat COVID-19, gene therapy has gained momentum to deal with viral infections in recent years (13). 

Among non-viral vectors, cationic polymer vectors, thanks to their distinguished characteristics, easy synthesis route, and ability to hybridize with different materials are under the spotlight for gene delivery studies (14). These vectors could facilitate the endocytosis of nucleic acid to cross the cellular and subcellular membranes by utilizing electrostatic condensation of the negatively-charged nucleic acids into the nano-scaled poly-cationic complex, forming positively charged polyplexes and, subsequently, enhance the cellular uptake of target cells (15).

Recently, metal-Organic framework (MOF) was presented as a new synthetic compound for carrying different biomolecules (16). These rapidly growing classes of microporous materials are built by coordination-driven self-assembly of metal ions and organic molecules as a ligand (organic linker species). Their physicochemical features including their self-assembly, porosity, tunability, size, biodegradability, biocompatibility, and functionalization capabilities, demonstrate a lack of toxicity and so forth has seen them be regarded as an interesting option for biomedical studies(8, 17, 18). Their pliable nature, high strength, and large surface area provide good flexibility for surface modification during synthesis or post-manufacture process (19, 20). 

With the ability to enclose biological molecules without changing their nature, MOFs, have a high loading capacity for drugs and any chemical substance which can protect them from environmental conditions such as enzymes (13, 21). Due to their unique abilities, they have been investigated in many studies in the field of gene therapy and diagnosis (13).

 Zirconium-based MOFs (Zr-MOFs), owing to their low toxicity, biocompatible characteristics, stable structure, and successful post-synthetic modification are one group of novel structures for the delivery of different therapeutic agents (14). Various studies have expressed the application of Zr-MOFs in different biological fields such as nucleic acids biosensors (DNA (22, 23), miRNA, and ATP (24)), co-delivery of Cisplatin and siRNAs (25), ssDNA delivery (26), biological gas storage and delivery, drug delivery (27), photodynamic (28), and nucleic acids delivery (15)(review). 

Polyethyleneimine (PEI) is a transfection agent with high pH buffering capacity that can protect the nucleotides from degradation and provide the possibility for endosomal escape, but its induced cytotoxicity is the major challenge of PEI application (29). Different study results confirmed that PEI with low molecular weight has a lower cytotoxicity and transfection efficiency than its high molecular weight variant. Thus, low molecular weight-PEI conjugated with other cationic carriers enhanced and improved transfection efficiency (29-32).

Herein we aimed to develop the polyethyleneimine (PEI) polymer grafted Zr-based MOFs as a gene delivery vector, so two types of Zr-based MOFs, namely NH_2_-UIO-66 and UIO-66, by post-modification with PEI10000 were fabricated, and their potential as a plasmid gene carrier was investigated on the A549 cell line.

## Materials and Methods

***Materials***

The A549 cell line (ATCC number CCL-185^TM^) has been bought from the Pasteur Institute of Iran. Dulbecco’s Modified Eagle’s Medium (DMEM) and Fetal Bovine Serum (FBS) were purchased from GIBCO (Gaithersburg, USA). Dimethyl sulfoxide (DMSO), 6-bromohexanoic acid, MTT dye (3-(4,5-dimethyl-2-thiazolyl)-2,5-diphenyl-2H-tetrazolium bromide), anhydrous methanol, Zirconium tetrachloride (ZrCl_4_), 1-Ethyl-3-(3-dimethyl aminopropyl) carbodiimide (EDC), and N-hydroxysuccinimide (NHS) have been acquired from Sigma–Aldrich (Germany). Branched polyethyleneimine (b-PEI 10 kDa) has been ordered from Polysciences Inc. (Warrington, PA, USA). EGFP encoding plasmid DNA was ordered from Promega (USA).

Terephthalic acid ligand and 2-aminoterephthalic acid ligand have been purchased from Merck (Germany). Ethanol 96%, N, N-dimethyl formamide (DMF), and Hydrochloric acid 30% (HCl) have been ordered from Merck (Germany).

***Synthesis of UiO-66*** ***MOF ***

**Figure 1:**
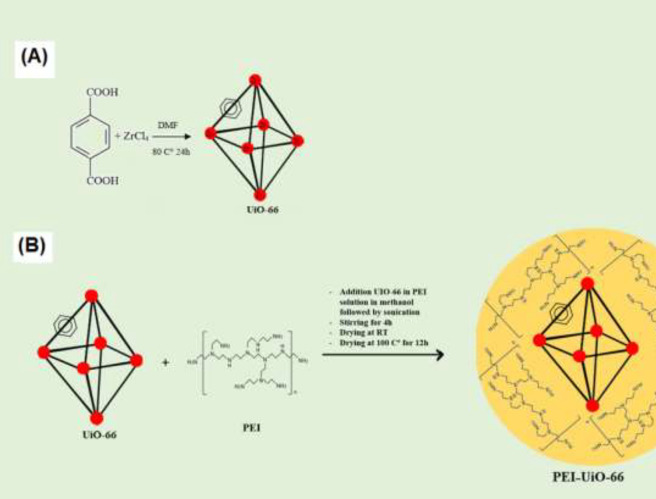
Schematic representation of the synthesis and modification of UiO-66 with PEI

The UiO-66 MOF structure was synthesized by a hydrothermal method according to the Katz *et al*. method (33). The synthesized route was similar to the former MOF’s structure with slight modifications using terephthalic acid (1,4-benzene dicarboxylic acid (BDC)) as a ligand. In this process, terephthalic acid ligand (1,4-benzene dicarboxylic acid (BDC)) (0.123 Gr, 0.75 mmol) and ZrCl_4_ (0.125 Gr, 0.54 mmol with 1 ml concentrated HCl) were mixed in 15 ml DMF. Then, the product was washed with 15 ml DMF (2X) and 15 ml ethanol (3X), and finally, it was placed in the oven at 100 °C overnight (Figure 1A).

***Synthesis of polyethyleneimine-conjugated MOF (PEI-UIO-66)***

UiO-66 powder (0.1 g) was added to a stirring solution of PEI (0.2 gin 2 ml anhydrous methanol) for 4 hr. The final solution was placed in the oven at 100 °C for 12 hr (Figure 1B).

***Synthesis of amine-functionalized MOF (NH***_2_ ***-UiO-66)***

**Figure 2:**
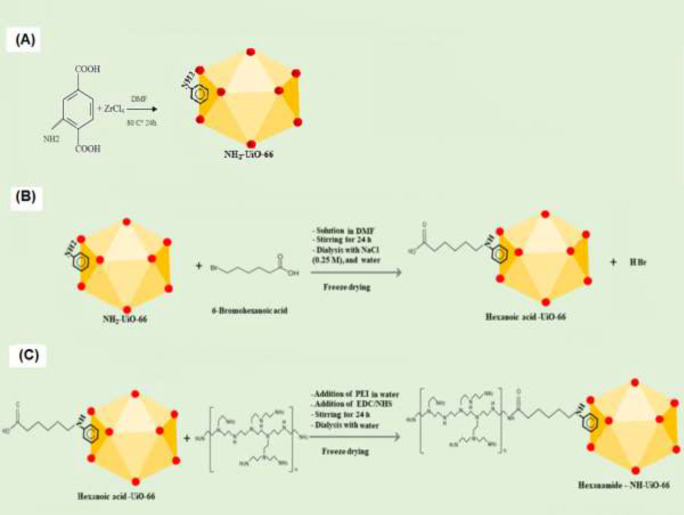
Schematic representation of the synthesis and modification of NH2-UiO-66

NH_2_-UiO-66 was also synthesized by a hydrothermal method according to Katz *et al*. (33). In this method 2-aminoterephthalic acid ligand (0.134 g, 0.75 mmol) was dissolved in 10 ml DMF, and ZrCl_4_ (0.125 g, 0.54 mmol) with 1 ml concentrated HCl was dissolved in 5 ml DMF under sonification for 20 min at RT. After mixing, the solution was filtered with Whatman paper. Then, the solution was transferred to a Teflon-lined autoclave and placed in an oven for 24 hr at 90 °C. Afterward, the product was washed with 15 ml DMF (2X) and 15 ml ethanol (3X), and finally, it was placed in the oven at 100 °C overnight (Figure 2A).

***Synthesis of polyethyleneimine hexanoic acid MOF (PEI-Hex-UIO-66)***

*A. Synthesis of Hex-UiO-66*

100 mg NH_2_-UiO-66 was dissolved in 2 ml DMF under sonification for 5 mins at RT. 6-bromohexanoic acid was dissolved in DMF, and the solution was added drop-wisely to a stirring solution of MOF in DMF at RT for 1 hr and continued for 24 hr. The resulting product was dialyzed once against NaCl (0.25 M) in a 12 kDa cut-off Spectra/Por dialysis tube for 4 hr and then against water for 48 hr. Finally, the product was dried under a freeze dryer (Martin Christ, Alpha 2–4 LD plus, Germany) (Figure 2B).

*B. Synthesis of PEI-Hex-UIO-66*

Hex-UiO-66 (20 mg) was dissolved in 10 ml DDW under sonification for 5 mins at RT. PEI0000 (500.85 mg), EDC (19.2 mg), and NHS (11.54 mg) were dissolved in 2 ml DDW and added drop-wisely to a stirring solution of Hex-UiO-66 at RT for 2 hrs. In order to remove unreacted solvents, the PEI-conjugated Hex-UiO-66 (PEI-Hex-UIO-66) was dialyzed against deionized water for 48 hr (the Spectra/Por dialysis membranes with MWCO of 12 kDa, Spectrum Laboratories, Houston, USA) followed by freeze-drying (Martin Christ, Alpha 2–4 LD plus, Germany) (Figure 2C).

***Characterization of nanoparticles***

The synthesized MOFs and PEI-grafted structures were characterized by X-ray Diffraction (XRD) (Philips’s analytical X-ray) and Fourier Transform Infrared Spectroscopy (FTIR) (Perkin Elmer, Spectrum GX, USA).

The morphology of the manufactured structures was evaluated by Scanning Electron Microscopy (SEM) and Transmission Electron Microscopy (TEM).

***Buffer capacity assay***

The buffering capacity of synthesized nanoparticles and PEI 10kDa, at an optimized concentration of 0.4 mg/ml, were evaluated by acid-base titration. The pH of the solutions was adjusted to 12 using 0.1 M NaOH, and, after that, 5 μl of 0.1 M HCl was subsequently added until the pH of solutions reached 2. The solution pH was measured with a pH meter (Mettler Todelo, Greifensee, Switzerland). The slope of the plot of pH versus the amount of HCl indicates the buffering capacity of the modified MOF structures. 

***Preparation of polyplexes (Complex of PEI-MOFs/pDNA)***

Different concentrations of PEI-MOFs nanoparticles (carrier (C)) were separately diluted into 50 μl of HBG buffer (HEPES-buffered glucose, 20 mM HEPES, 5% glucose, pH 7.4) and added to 50 μl solutions of the plasmid (pEGFP) (P) in the same solvent (4 μg/50 μl), at different weight ratios (C/P) and mixed. Then, they were incubated at room temperature for 20 min to form polyplexes (polycation/DNA complexes). The prepared C/P ratios were at 1, 2, 4, and 6 (weight/weight ratios).

***Size and zeta potential investigation***

The zeta potential and size distribution of complexes were measured with Zetasizer Nano ZS (Malvern Instruments, UK) at a C/P ratio of 4. Different amounts of PEI-grafted MOFs were diluted in 125 μl of HBG buffer and mixed with an equal volume of the same buffer containing DNA with a final DNA concentration of 5 μg/ml. The mixture was incubated for 20 min at room temperature. The measurements were performed three times for each sample, and the results are presented as mean ± SD.

***Gel retardation assay***

The capability of PEI-Hex-UiO-66 and PEI-UiO-66 for DNA condensation was investigated through the agarose gel retardation assay. Polyplexes of PEI-modified MOFs (PEI-Hex-UiO-66 and PEI-UiO-66) and PEI 10kDa at different C/P ratios of 1.0, 2.0, 4.0, and 6.0 were prepared and used for the gel retardation assay.

Gel was prepared with Agarose (1%, w/v) and green viewer (0.1 μl/ml) (Biotium, Fremont, CA, USA) in TBE (Tris-borate-EDTA) buffer (1X). After loading the samples, electrophoresis was performed at 70 V for 45 min, and then, the gel documentation Imaging System (Gel Doc) was used for DNA band observation.

***Cell culture***

Lung cancer cells (A549) were cultured in DMEM (Dulbecco’s Modified Eagle’s Medium) high glucose media that was augmented with 10% FBS (Fetal bovine serum) and 1% antibiotic (penicillin/streptomycin) and incubated in a humidified atmosphere (under) with 5% CO2 at 37 °C. 

***In vitro transfection assay***

Cells were seeded in 96-well plates (10000 cells/well) and incubated for 24 hr. Next, the media was removed and exchanged with 100 μl of fresh media (without FBS). Polyplexes were added to the cells in three C/P ratios of 2, 4, and 6. PEI 10000 Da at the same C/P ratio was used as a control. After 4 hr, the medium was exchanged with fresh complete growth media (with 10% FBS) and incubated for 24 hrs. For transfection assay, the medium was discarded, and 50 μl of lysis buffer solution was added to each well. 30 μl of the lysis cells were placed on a fluorescent plate and measured by fluorimetry, and the percentage of transfected cells was determined using a fluorescent plate reader (Victor X5, PerkinElmer, USA). The GFP fluorescence measurement was performed at 485 nm and 535 nm bandpass filters.

***Cell viability assay***

The cells were seeded at a density of 10,000 cells/well in 96-well micro-assay plates and incubated for 24 hr. The prepared polyplexes (at the same ratio as the transfection assay) were added to the cells. The assay was performed according to the transfection assay. After 24 hr of incubation, 10 μl of MTT (3-(4, 5-dimethylthiazol-2-yl)-2,5-diphenyltetrazoliumbromide) solution (5 mg/ml in PBS) was added to each well and incubated at 37 °C. For MTT evaluation, the medium was aspirated off, and 100 μl of DMSO solution was added to each well. The absorbance of each well was measured at 570/630 nm. The metabolic activity percentage of the polyplex-treated cells was calculated relative to the untreated cells as a control.

***Cellular uptake evaluation***

The uptake of polyplexes was investigated on the A549 cell line using flow cytometry. Polyplexes of modified MOFs were prepared at a C/P ratio of 4 and PEI 10000 kDa was used as a control to validate the transfection assay.

Cells were cultured in 24-well plates (100,000 cells/well) and incubated for 24 hr. The polyplexes were added to cells in an optimized C/P ratio and incubated in a situation similar to the transfection protocol. Then, cells were collected, rinsed with PBS, and analyzed by flow cytometry. The data of flow cytometry was analyzed using the FlowJo program.

***Statistical analysis***

The data was statistically analyzed using one-way ANOVA with a *P*-value<0.05 or less as the significance level. Values are represented as mean ± standard deviation (SD) triplicates.

## Results

***Preparation and characterization of nanoparticles***

*Chemical structural analysis*

**Figure 3:**
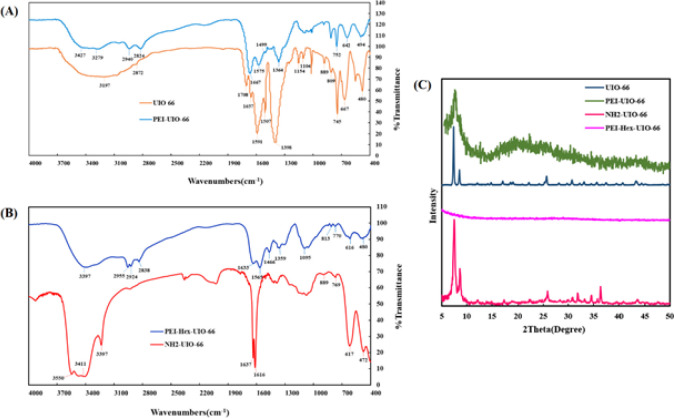
A) FTIR spectra of PEI-UIO-66 and UIO-66, B) PEI-Hex -UIO-66 and NH2-UIO-66, and C) XRD patterns of PEI-UIO-66, UIO-66, PEI-Hex-UIO-66, and NH2-UIO-66

The chemical composition of MOFs was determined through FTIR analysis, and the corresponding results are presented in Figure 3 (A, B). According to previous studies, the results obtained showed that UIO-66 has absorption peaks at 481 (34, 35), 1398, 1507, 1591 (34), and 3440/3200 (36) cm^−1^ which related to Zr-O vibration on Zr-based MOFs, C=C bond in the benzene ring, O-C-O asymmetric/symmetric stretching, and O-H groups, respectively.

After the post-synthetic modification of UiO-66 particles with PEI, the presence of PEI on UIO-66 (PEI-UIO-66) with clearly representative peaks at 3427/3279, 2940/2824, 1575/1364, 1467, and 1088/1015 cm^−1^ was shown that correspond to -NH2/-NH bending, asymmetric/symmetric –CH_2_ stretching, C-N stretching, and N-H wagging of PEI, respectively (Figure 3A) (36, 37).

The results of PEI-Hex-UIO-66 FTIR analysis shows that the 2956/2824 and 3397 cm^−1^ signals are related to the CH2 vibration stretching and unreacted (free) amino groups, respectively (38). 

In NH_2_-UIO-66, the peaks at 3236 and 3550/3411 cm^−1^ are assigned to the NH2 groups (38, 39). 

In both methods, peaks appeared or became stronger in the range of 2800–3000 after adding PEI, which were related to the presence of PEI on modified MOFs (Figure 3B) (38). 

The peaks under 800 cm^-1^ (35) corresponded to Zr-O vibration on Zr-based MOFs. Finally, matched peaks have confirmed the presence of PEI in modified MOFs.

The structure and crystallinity of the synthesized UiO-66-NH2 and UIO-66 were assessed using X-ray diffraction (XRD) analysis. The XRD pattern revealed the presence of two distinct peaks at 2θ values of 7.3° – 7.4° and 8.4° – 8.5°. These peaks are indicative of the characteristic diffraction pattern associated with the UiO-66-based structure. The presence of these specific peaks in the XRD pattern confirms the successful synthesis of the UiO-66-based structure. These peaks correspond to the specific crystallographic planes and lattice spacing of the UiO-66 framework. The appearance of these peaks at the expected 2θ values provides evidence for the formation of the desired UiO-66 structure in both UiO-66-NH2 and UIO-66 samples. By analyzing the XRD pattern and identifying these characteristic peaks, we can conclude that the synthesized materials possess the desired UiO-66-based structure with the expected crystallinity. This information is crucial for understanding the structural properties and potential applications of the synthesized UiO-66-based materials (34). 

After the post-synthetic modification of UIO-66 and UiO-66-NH2 with PEI, the XRD analysis of the resulting PEI-UIO-66 sample revealed the presence of the main peak associated with UIO-66. However, the intensity of this peak was observed to be weak compared to the original UIO-66 sample. This decrease in intensity indicates a decline in crystallinity for UIO-66 after the modification. Despite the decrease in crystallinity, it is important to note that the structure of UIO-66 remained intact even after the post-synthetic modification with PEI. This means that the overall framework and connectivity of the UIO-66 structure were preserved. The presence of the main peak in the XRD pattern of PEI-UIO-66 confirms that the essential structural features of UIO-66 were not significantly altered or disrupted by the modification process. The decline in crystallinity observed in PEI-UIO-66 suggests that the introduction of PEI into the UIO-66 framework may have caused some disorder or disruption in the arrangement of the crystalline lattice. However, the fact that the main peak is still present indicates that the fundamental structure of UIO-66 remains intact, albeit with reduced crystallinity (40). In the XRD analysis of the PEI-UIO-66 sample, a broad peak was observed between 14° – 30°. This peak can be attributed to the presence of free PEI, which exhibits an amorphous structure. The appearance of this broad peak suggests that some PEI molecules were not incorporated into the UIO-66 framework during the post-synthetic modification process (41). In PEI-Hex-UIO-66, the absence of the peaks related to UiO-66 indicated the presence of PEI-Hex attached to the UIO-66 that would interfere with the structural pattern, which destroyed the MOF peaks after modification and loss of crystallinity (Figure 3C).

*Morphological analysis*

The morphological analysis of PEI-UIO-66 nanoparticles exhibited evenly distributed particles with a spherical morphology and particle size that was approximately 100 nm. 

**Figure 4:**
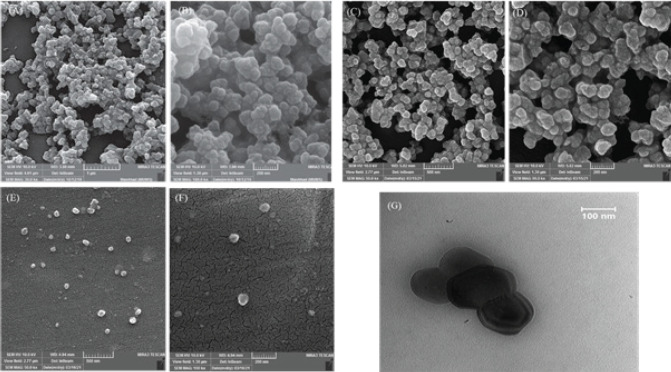
Morphological analysis of PEI-HEX-UIO-66 and PEI-UIO-66 was performed with SEM and TEM imaging

The agglomeration was observed in the case of PEI-HEX-UIO-66, forming three-dimensional (3D) nano-spherical clusters with heterogeneous size distribution. For a better study of the structure, the TEM imaging of NH_2_-UiO-66 nanoparticles was utilized, which demonstrated triangular base-pyramid shape of the structure maintenance during the PEI addition process (Figure 4).

***Determination of size and zeta potential of synthesized vectors***

The particle size and zeta potential of modified and unmodified MOFs are shown in Table 1. 

***Buffer capacity assay***

**Figure 5:**
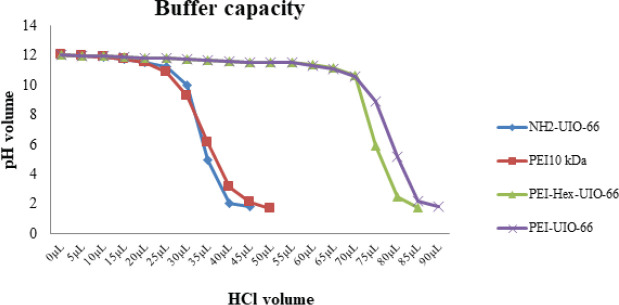
Buffering capacity of different vectors of PEI 10000 Da, PEI-HEX-UIO-66, and PEI-UIO-66, NH2-UiO-66

The effect of the added primary amines by PEI grafting on the buffering capacity of MOFs was estimated by measuring the change in pH of the polymer solution (0.4 mg/ml) upon titration with 1M HCl (Figure 5) which was evaluated based on the presence of primary amines in the chemical structure of vectors and their behavior in acidic conditions, which could be used as biomimicry for the proton sponge effect of PEI. 

The results showed that unmodified MOF and PEI 10000 Da had a lower buffering capacity than PEI 10000 Da grafted MOFs (Figure 5).

***Gel retardation assay***

**Figure 6:**
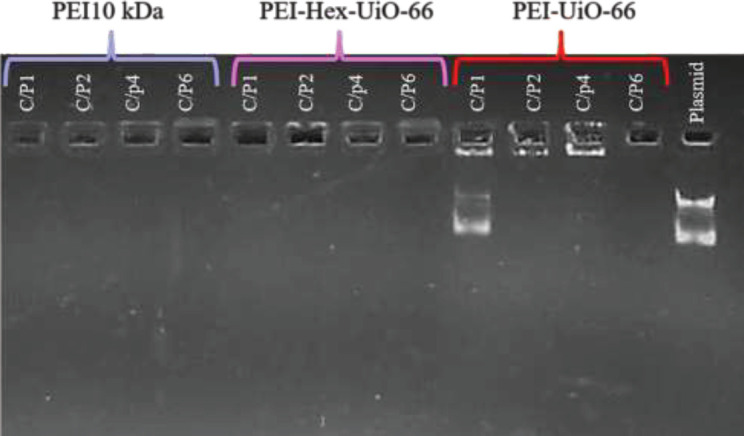
Gel retardation assay of polyplexes (PEI 10kDa, PEI-HEX-UIO-66, and PEI-UIO-66) that were prepared at different C/P ratios (1, 2, 4, and 6)

Figure 6 shows the results of each carrier’s ability to condense DNA. According to the results, at a C/P ratio of 2, all nanoparticles completely condensed the plasmid DNA. In addition, PEI-Hex-UiO-66 could condense the plasmid DNA at all C/P ratios as well as PEI 10kDa*.*

***Transfection efficiency of vectors***

**Figure 7:**
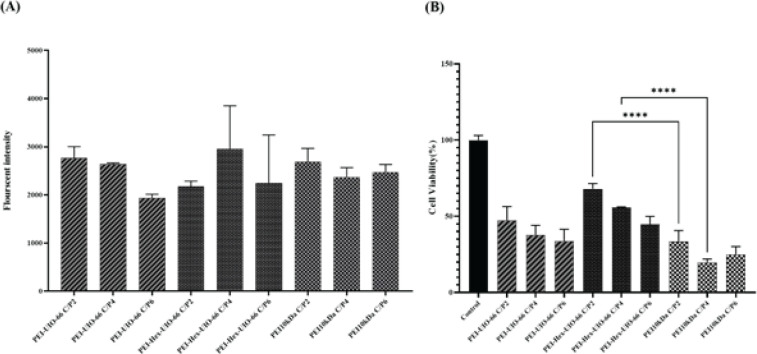
A) Transfection efficacy of PEI-Hex-UIO 66, PEI-UIO 66, and PEI 10kDa polyplexes at different C/P ratios (2, 4, and 6) in A549 cells (mean ± standard deviation (SD) of triplicates), and B) Metabolic activity of A549 cells after treatment with PEI-Hex-UIO 66, PEI-UIO 66, and PEI 10kDa polyplexes at different C/P ratios (2, 4, and 6) determined by MTT assay (mean ± standard deviation (SD) of triplicates). **** *P*-Value<0.0001 PEI-Hex-UIO-66 compared to PEI 10kDa at C/P ratios of 2 and 4

The transfection efficiency of modified MOFs was investigated on the A549 cell line. The cancer cells were transfected with different ratios of vectors/DNA (C/P ratios of 2, 4, and 6) which were selected based on the gel retardation assay results. The results showed no significant difference between modified MOFs with PEI 10 kDa (Figure 7A). 

***Cell viability of vectors***

The cell viability of A549 cells treated with carrier/pDNA complexes (polyplexes) was evaluated using the MTT assay (Figure 7B). The C/P ratios of prepared polyplexes were selected similar to the transfection assay.

As shown in Figure 7, the highest metabolic activity had been observed for PEI-Hex-UiO-66 polyplexes at C/P ratios of 2 and 4, even less toxic than PEI 10kDa. 

***Cellular uptake evaluation***

**Figure 8:**
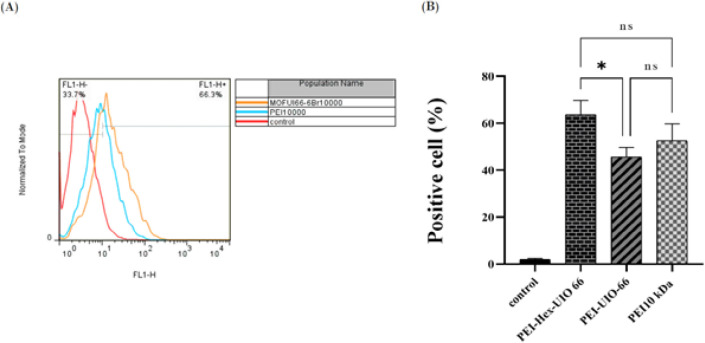
Cellular uptake of PEI-Hex-UIO 66, PEI-UIO 66, and PEI 10kDa at C/P4 determined by flow cytometry (Mean ± SD, n = 3)

The cellular uptake of modified MOFs and PEI 10kDa was investigated by flow cytometry at C/P ratio of 4, and the percentage of the EGFP-positive cells and relative fluorescence intensity was determined (Figure 8). The results showed that polyplexes of PEI-Hex-UiO-66 and PEI-UiO-66 with average percentages of 68% and 45% had higher and lower cellular uptakes, respectively. 

PEI-Hex-UiO-66 showed better cellular uptake compared to PEI-UiO-66 (*P*-value≤0.05). However, the differences with PEI 10kDa were not significant.

A) Synthesis of UiO-66, and B) Synthesis of PEI-UIO-66

PEI: Polyethyleneimine, PEI-UIO-66: Polyethyleneimine-conjugated MOF

A) Synthesis of NH2-UiO-66, B) Synthesis of Hex-UiO-66 through reacting with 6-bromohexanoic acid, and C) Synthesis of PEI-Hex-UIO-66 through reacting with PEI.

PEI: Polyethyleneimine, PEI-Hex-UIO-66: Polyethyleneimine Hexanoic Acid MOF

PEI-UIO-66: Polyethyleneimine-conjugated MOF, PEI-Hex-UIO-66: Polyethyleneimine Hexanoic Acid MOF

A & B) SEM image of NH2-UIO-66, C & D) SEM image of PEI-HEX-UIO-66, E & F) SEM images of PEI-UIO-66, and G) TEM image of PEI-HEX-UIO-66.

PEI-Hex-UIO-66: Polyethyleneimine Hexanoic Acid MOF, PEI-UIO-66: Polyethyleneimine-conjugated MOF

**Table 1:** Size and zeta potential of MOFs and MOFs/pDNA complexes at C/P ratio of 4 (w/w) (mean ± standard deviation, n=3)

**Nanoparticle**	**Size (nm)**	**Polydispersity index (PDI)**	**Zeta potential (mv)**
**NH**_2_ **-UiO-66**	157.5 ± 22.4	0.386 ± 0	+ 21.2	
**PEI-Hex-UIO-66**	366.8 ± 5.1	0.253 ± 0.03	+ 32.3 ± 1.1	
**pDNA/PEI-Hex-UIO-66**	296.4 ± 27.5	0.35 ± 0.01	+ 25.1 ± 1.7	
**UiO-66**	201.3 ± 0	0.294 ± 0	- 0.95± 0.07	
**PEI-UIO-66**	285.9 ± 9.5	0.33 ± 0.01	+ 18.8 ± 2.03	
**pDNA/PEI-UIO-66**	240.6 ± 44.4	0.35 ± 0.06	+ 17.03 ± 0.25	

MOFs: Metal-Organic Frameworks, MOFs/pDNA: Metal-Organic Frameworks/plasmid DNA, C/P: carrier/plasmid, PEI-UIO-66: Polyethyleneimine-conjugated MOF, pDNA/PEI-UIO-66: plasmid DNA/PEI-UIO-66, PEI-Hex-UIO-66: Polyethyleneimine Hexanoic Acid MOF, pDNA/PEI-Hex-UIO-66: plasmid DNA/ PEI-Hex-UIO-66

PEI-Hex-UIO-66: Polyethyleneimine Hexanoic Acid MOF, PEI-UIO-66: Polyethyleneimine-conjugated MOF

PEI 10kDa: Polyethyleneimine 10000 Da, PEI-Hex-UIO-66: Polyethyleneimine Hexanoic Acid MOF, PEI-UIO-66: Polyethyleneimine-conjugated MOF, C/P: carrier/plasmid

PEI 10kDa: Polyethyleneimine 10000 Da, PEI-Hex-UIO-66: Polyethyleneimine Hexanoic Acid MOF, PEI-UIO-66: Polyethyleneimine-conjugated MOF, C/P: carrier/plasmid.

A) Representative histogram of A549 cell line transfected by PEI-Hex-UIO 66, PEI-UIO 66, and PEI 10 kDa, and B) Comparison of different groups. * P-value<0.05, PEI-Hex-UIO66 compared to PEI-UIO66 and NS non-significant, modified MOF compared to PEI 10 kDa at C/P ratio of 4.

PEI 10kDa: Polyethyleneimine 10000 Da, PEI-Hex-UIO-66: Polyethyleneimine Hexanoic Acid MOF, PEI-UIO-66: Polyethyleneimine-conjugated MOF

## Discussion

In gene therapy, developing highly efficient gene delivery carriers with non-toxic properties has remained a challenge (6, 42).

MOFs are new materials in the bio-molecule delivery approach whose applicable features attract researchers’ attention. However, the usage of MOFs as a vector to transport biomolecules such as proteins, DNA, and RNA is still in its infancy (1). The use of MOFs as a gene carrier is a less common choice due to its low loading efficiency of high molecular weight molecules. Most of the studies have been done on small-size nucleic acids such as siRNA, and few studies have reported pDNA delivery, including two that reported a delivery system in which pDNA was inserted during ZIF-8 synthesis (1, 43, 44). 

Modification and functionalization of the MOF surface, such as the use of capping molecules during MOF synthesis and covalent and non-covalent post-synthesis modification, is one of the fruitful approaches to extend their application in biomedical studies (16). By surface modification and placing the gene cargo on the MOF surface, the limitations of encapsulating nucleic acids with large sizes, especially pDNA, could be overcome. In a recent study, PEI was used to modify the surface of MIL-100 (Fe) for use in gene transfer. Ringaci *et al*. presented a suitable carrier for co-delivery by placing the protein agent inside MOF and loading pDNA on the surface of this carrier (43). Researchers used encapsulation of carbonized chitosan (CTS) inside zif8 to produce hierarchical mesoporous carbon (MPC), which increased the surface and efficiency of material loading on zif8. Additionally, by adding cell-penetrating peptides to MPC, its efficiency in the cellular uptake of pDNA and oligonucleotides was increased (45). Modification of MOF’s surface with polymers made it possible to co-deliver both plasmid and drug (21). 

In our study, we seek to introduce a new carrier based on the zirconium-based metal-organic framework with more acceptable properties for gene delivery studies. Accordingly, to increase the condensation potential of synthesized MOFs, their surfaces were modified by grafting PEI in order to exploit its cationic feature and proton buffering capacity (46).

Two different approaches were considered to modify two types of Zr-based MOFs with PEI 10 kDa; firstly, PEI coated UIO-66 (PEI-UIO-66); in the second approach, the polymer was linked to NH_2_-UIO-66 (PEI-Hex-UIO-66). The PEI-UIO-66 was developed through the wet impregnation method.

The functionalization of PEI-Hex-UIO-66 was performed through the EDC/NHS + 6-bromohexanoic acid conjugation method, and the PEI 10k Da was linked to NH_2_-UIO-66. Several studies used this approach to link PEI to different molecules via its primary amino groups (47-50).

FTIR analysis of synthesized MOFs confirmed the presence of associated functional groups in both wet impregnation and covalent linking approaches, indicating that PEI was successfully linked to MOFs. The XRD analysis revealed that the crystalline structures of both UIO-66 and NH2-UIO-66 exhibited a strong consistency with the simulated pattern, providing compelling evidence for the successful synthesis of these intricate MOFs (34, 36, 51).

The inclusion of PEI in the PEI-UIO-66 composite had notable effects on its XRD pattern. Specifically, the peaks in the XRD spectrum became broader and their overall intensity decreased. This observation indicates that the crystallinity of the UIO-66 material experienced a reduction after the modification process involving PEI. Despite this reduction in crystallinity, it is essential to note that the fundamental structure of the UIO-66 remained largely unchanged. The modification with PEI did not lead to a significant disruption of the underlying framework or overall arrangement of the UIO-66 material. This suggests that while the crystalline nature of the material was affected, the core structural integrity of the UIO-66 framework was preserved (40, 41).

From a chemical and physical standpoint, XRD analysis of PEI-Hex-UIO-66 presented intriguing findings. Notably, the absence of any discernible peaks in the XRD spectrum is a strong indication of significant changes in the material’s crystalline structure. Moreover, the characteristic peaks that were previously attributed to the MOF had vanished. These outcomes were remarkably reminiscent of a prior study, which demonstrated the successful binding of PEI to the MOF surface. This binding process appeared to extend throughout the entire surface of the MOF, effectively encapsulating it with the PEI molecules. The disappearance of the MOF’s peaks in the XRD analysis implied that the structural arrangement of MOF underwent substantial modification due to the introduction of PEI. It’s reasonable to infer that the PEI-Hex molecules, in their endeavor to cover the MOF surface, might have induced disorder in the original crystalline structure, leading to the loss of the characteristic diffraction peaks. This phenomenon could be attributed to the interference caused by the presence of PEI-Hex, potentially altering the way X-rays interact with the material. The congruence between the results of this study and the earlier one supported the notion that PEI-Hex has indeed effectively covered the MOF, leading to a profound change in the material’s XRD signature (14). 

Morphology analysis with transmission electron microscopy (TEM) imaging was used for a further structural study of PEI-Hex-UIO-66. The resulting image shows that the triangular base-pyramid structure of NH_2_-UiO-66 was preserved during the PEI addition process

Furthermore, scanning electron microscopy (SEM) imaging showed these nanoparticles have a spherical or quasi-spherical shape. The geometric features of nanoparticles are effective factors in their cellular uptake and cell internalization. For example, Herd *et al*. (2013), in the study of cellular uptake, reported three silica NP shapes; geometrically, the spherical shapes have the highest capability for cellular uptake compared to the rod and cylindrical forms(52).

The size of polyplexes is another substantial factor in cellular uptake and successful gene delivery (53) (54). 

Based on the dynamic light scattering (DLS) results, the polyplex formation leads to a decrease in particle size and surface charge of nanoparticles that show successful DNA loading and condensation. This phenomenon was observed in previous studies, which demonstrated that the presence of polymer in nanoparticle structure enhanced the ability of condensation with negatively charged plasmid and decreased polyplex size (14).

A study reported that the DNA payload led to coverage of all surface pores on MOFs’ structure, and the colloidal and dispersity properties of MOF in physiologic liquids were superior and prevented particles from aggregation (55).

In gene delivery, the gene condensation capability of the carrier is a critical factor that is related to the amount of positive charge on the carrier (42). The results of the gel retardation assay showed that PEI-UIO-66 and PEI-Hex-UIO-66 could completely condense the plasmid. However, due to the smaller surface charge of the PEI-UIO-66, it was not able to condense the plasmid at a C/P ratio of 2. The presence of unsaturated coordination sites, which may occur after activation of UiO-66, could be another reason that affects the condensation ability of PEI-UIO-66.

Adding cationic materials and polymers to the MOF surface increases their surface charge, which can increase the electrostatic interaction between the nucleic acid (negatively charged) and the modified MOF (positively charged) resulting in increased transfection efficiency (13, 14). Cationic polymers condense the DNA and protect it against serum and intracellular enzymes due to the positive surface charge of the resulting polyplex (56). Doug *et al.* used the addition of polyamine PGMA-EA to modify NH2-UIO-66, and the resulting nanocarriers were successful in plasmid delivery and mRNA delivery in subsequence studies which done by Sun et al. (14, 15). 

One of the main problems for efficient gene delivery is degradation of the genetic materials in endo/lysosomal compartments by acid hydrolases. PEI can provide early escape of genetic materials from endosomes into the cytosol before starting the degradation process via proton sponge activation (57). The mechanism performed by the efflux of chloride ions against the high amine content of PEI (or cationic ions of other polymers in the vesicle) leads to increased osmotic pressure of the vesicle followed by over-absorbing water disrupting the vesicle membrane (57, 58). Therefore, the buffering capacity of MOFs is an important factor that affects their efficiency. The results of the buffering capacity assay showed that the buffering capacity of modified MOFs was higher than unmodified NH_2_-UIO-66 and PEI 10 kDa. In other words, the conjugation of MOF with PEI has a synergistic effect on buffer capacity. 

For efficient delivery of different nucleic acid cargoes, such as various types of RNAs and DNAs, to the cytoplasm and nucleus compartment, there are many barriers that should be overcome (59). In general, the cellular uptake of nanoparticles depends on the cell type and physicochemical properties of nanoparticles (shape, size, surface charge, and stiffness) (60, 61). The flow cytometry results indicated that PEI-Hex-UIO-66 delivered the pDNA to the nucleus more efficiently than PEI-UIO-66. The reason could be the complete covering of MOF (NH_2_-UIO-66) with PEI, which equals more DNA loading on the MOF surface. Furthermore, the benefit of PEI-Hex-UIO-66 could be due to the effect of Zr on the endosome membrane by interacting with the negative charge of the membrane and disrupting it, which leads to better escape from the endosome. The other reason may be the interaction of Zr with cytosolic phosphate and the degradation of the MOF, followed by the release of DNA inside the cells (62). It is shown that nanoparticles with a size of less than 200 nm are likely absorbed by endocytosis, while larger particles are intended to enter from the micropinocytosis pathway (60). Recent studies have proposed more than one mechanism for the delivery of nanoparticles (60, 61, 63). In a study, on the effect of SiO_2_ nanoparticles’ sizes on their uptake pathways in the A549 cell line, it was observed that nanoparticles with the size of 50 and 100 nm were uptaken by the cell through caovalin and clathrin pathways, and the 300 nm SiO_2_ nanoparticles entered the cell through micropinocytosis pathway with less efficiency compared to other nanoparticles (64). It is expected that the nanoparticles synthesized in our study enter the cell through the micropinocytosis pathway.

Generally, cationic nanoparticles with high positive surface charge density (such as nanoparticles that are derived from PEI or have PEI in their structure) could interact with the cell membrane, leading to its permeability and perforation. Despite increasing their efficiency in gene delivery, this can cause them to be toxic to cells (61, 65). As observed in viability assays, increasing the concentration of nanoparticles is associated with an increase in toxicity. The MOFs toxicity evaluation showed that PEI-Hex-UIO-66 had the lowest toxicity relative to PEI-UIO-66 and PEI, respectively, which could be due to the lower amount of PEI content in PEI-Hex-UIO-66. In this study, PEI-HEX-UIO-66 nanoparticles with 63.7% cellular uptake showed the same results as in the study by Ringaci *et al*. (43).

PEI-HEX-UIO-66 nanoparticles had the highest transfection ability and the lowest toxicity compared to PEI-UIO-66, which indicates the superiority of this method in successfully modifying MOF and its application in gene delivery. Moreover, lower molecular weight or modified PEIs can be used to increase transfection efficiency and reduce toxicity. As in previous studies, other polyamines such as PGMA-EA were used for this purpose to modify MOF, and they were successful in the transfection of plasmid and mRNA (14, 15).

## Conclusion

In this study, we used two different methods to modify MOF by grafting PEI on the surface. In both methods, the PEI has been successfully placed on MOFs without any apparent deficiency in the MOFs’ backbone. The grafting of PEI on the NH_2_-UiO-66 showed a better result by being more effective in gene delivery and reducing toxicity than the wet impregnation method.

To increase efficiency and reduce toxicity, lower molecular weights or other modified PEI with lower positive charge could be used. It is suggested that this nanoparticle can be used in mRNA delivery systems. Also, because of its natural porous core, it can be used for drug delivery or co-delivery of drugs and nucleic acids.

## Authors’ Contributions

R KO, A H, M K, and M D contributed to the conception and design of the work. S K, L G, and A H contributed to the acquisition, analysis, and interpretation of data. SK prepared the manuscript draft. R KO and L G finalized the manuscript. All authors have read and agreed to the published version of the manuscript.

## Conflicts of Interest

The authors declare that they have no known competing financial interests or personal relationships that could have influenced the work reported in this paper.
